# Timely AVF creation in late-referral hemodialysis patients, a two-cohort study: Too late to plan, not too late to act

**DOI:** 10.1177/11297298251390107

**Published:** 2025-11-09

**Authors:** Elettra Lomeo, Roberto Mangiacapra, Francesca Maria D’Ascenzo, Raghvinder Pal Singh Gambhir, Mitta Nivedita, Giuseppe Grandaliano, Domenico Valenti, Francesco Pesce

**Affiliations:** 1 Nephrology, Dialysis and Transplantation Unit, Department of Medical and Surgical Sciences, Fondazione Policlinico Universitario A. Gemelli IRCCS, Rome, Italy; 2 Department of Vascular Surgery, King’s College Hospital, London, UK; 3 Department of Translational Medicine and Surgery, Universita’ Cattolica del Sacro Cuore, Rome, Italy; 4 Division of Renal Medicine, Ospedale Isola Tiberina-Gemelli Isola, Rome, Italy

**Keywords:** CVC, CRBSI, early AVF, late referral

## Abstract

**Background:**

Timely vascular access creation in end-stage kidney disease is critical for optimizing patient outcomes. While early nephrology referral (⩾90 days pre-dialysis) improves access patency and survival, late referral is associated with worse outcomes. Healthcare system structure and demographics may impact vascular access strategies. This study compared late-referral hemodialysis patients from two high-volume hospitals in different healthcare systems, focusing on vascular access and survival.

**Methods:**

This retrospective, two-center study included 463 late-referral hemodialysis patients (mean age 60.4 ± 15.8 years; 63.3% male) initiating dialysis with a tunneled CVC between January 2020 and May 2024 at King’s College Hospital, London (*n* = 249) and Policlinico A. Gemelli, Rome (*n* = 214). Primary outcome: all-cause mortality. Secondary outcomes: AVF creation, primary and secondary AVF patency, and CRBSI.

**Results:**

AVF recipients had significantly lower mortality than non-AVF patients (*p* = 0.002), and a lower bacteremia rate (*p* = 0.05) across both Italy and UK cohorts (*p* = 0.005 and *p* = 0.0005, respectively). Time to AVF creation was significantly associated with mortality (OR 1.003; 95% CI 1.000–1.006; *p* = 0.02). AVF creation was achieved in 42.1% (*n* = 195), with primary patency rate of 62.7% at 6 months. Ethnicity (*p* = 0.01) and age (*p* = 0.0004) influenced AVF type selection. Among non-AVF patients, 26% declined surgery despite eligibility, showing reduced survival (*p* = 0.03). CRBSIs occurred in 16% of patients, mostly Gram-positive (70.3%). Relapse rate was higher with CVC removal/replacement than guidewire exchange (19.4% vs 0%; *p* = 0.01).

**Conclusions:**

Early AVF creation in late-referral hemodialysis patients improves survival and reduces CRBSI risk. Reducing CVC requirement, especially in AVF-eligible patients, is a key strategy to improve outcomes.

## Introduction

Chronic kidney disease (CKD) is a growing global health burden, with an increasing number of patients progressing to end-stage kidney disease (ESKD) and requiring renal replacement therapy (RRT), primarily hemodialysis (HD).^1–3^ The choice of vascular access plays a critical role in patient outcomes, influencing infection rates, long-term survival, and healthcare costs.^4^ The 2019-Kidney Disease Outcomes Quality Initiative (KDOQI) guidelines replaced the “fistula first” paradigm with the “ESKD Life-Plan” which aims to ensure “the right access, for the right patient, at the right time.”^5^ While this approach is particularly relevant for elderly, cancer or near-term transplant patients, AVFs still provide lower infection risk, higher patency rates, and superior long-term outcomes compared to central venous catheters (CVCs).^6–9^

However, despite these recommendations, a substantial proportion of patients initiate HD with a CVC.^5,10^ In the United States, nearly 80% of incident HD patients start dialysis with a CVC, a trend observed in many other healthcare systems.^4^ This reliance on CVCs is associated with increased morbidity and mortality, largely due to catheter-related bloodstream infections (CRBSIs), thrombosis, and vascular stenosis.^11–16^

Late referral to nephrology, defined as an initial consultation occurring less than 90 days before dialysis initiation, is a key contributor to CVC dependence.^10,17^ Late-referred patients have higher mortality rates, increased hospitalizations, and a reduced likelihood of timely AVF creation, leading to prolonged CVC use.^10,18–20^ Indeed, early referral allows for vascular access planning and comorbidity optimization, whereas late-referral patients miss this opportunity due to delayed diagnosis, healthcare system inefficiencies, or patient-related factors.^12,21,22^ Notably, vascular access management strategies differ across healthcare systems, influenced by surgical expertise, hospital infrastructure, infection control protocols, and referral pathways.^10,17,23,24^

This study aims to evaluate the outcomes of late-referral HD patients in two high-volume hospitals with distinct healthcare systems: King’s College Hospital, London (UK), and Policlinico A. Gemelli, Rome (Italy). By evaluating vascular access selection, primary and secondary AVF patency, CVC-related infections, and patient survival, this analysis seeks to identify key factors influencing vascular access outcomes and highlight opportunities for optimizing the management of late-referred patients.

## Methods

### Study design and data collection

We performed a retrospective, observational, two-cohort study including late-referral patients managed at the nephrology and dialysis units of Policlinico A. Gemelli Foundation in Rome, Italy, and the nephrology and vascular unit of King’s College Hospital in London, United Kingdom between January 2020 and May 2024 who underwent HD after tunneled CVC placement. The primary endpoint was all-cause mortality in late referral patients. Secondary endpoints included AVF creation, primary and secondary AVF patency rate, and CRBSI incidence. We retrospectively collected clinical and demographic data from electronic medical records and surgical logs at both institutions, with cross-verification by two independent reviewers to ensure accuracy and consistency. Collected variables included demographic features (age, gender, ethnicity), clinical data (underlying causes of CKD, comorbidities, CVC-related complications, and treatments), and surgical details (AVF type creation, primary and secondary AVF patency).

### Study population

Eligible patients were: late-referral individuals, defined as those referred to nephrology care less than 3 months prior to initiating kidney replacement therapy, who started hemodialysis via a tunneled CVC and were aged over 18 years. Exclusion criteria encompassed patients with long-term CVC placement due to AVF or arteriovenous graft (AVG) malfunction or peritoneal dialysis patients converted to HD for catheter-related peritonitis. The study population included an Italian and a UK cohorts.

### Statistical analysis

Continuous variables were assessed for normality using the Shapiro-Wilk test. Normally distributed variables were summarized as means ± standard deviations (SD), while non-normally distributed variables were reported as medians with interquartile ranges (IQR). For between-group comparisons, we employed the Wilcoxon rank-sum test for non-normally distributed continuous variables due to its robustness against skewness, while we used the Student’s *t*-test for normally distributed data where applicable. Categorical variables were expressed as frequencies and percentages, with group differences evaluated using the Chi-square test or Fisher’s exact test when expected cell counts were low (<5). Survival analyses utilized the Kaplan-Meier method to estimate survival probabilities, with differences between survival curves assessed via the log-rank test. All statistical tests were two-tailed, and results were accompanied by relevant test statistics and 95% confidence intervals where appropriate to enhance interpretability. A *p*-value threshold of less than 0.05 was considered indicative of statistical significance. We performed statistical analyses using IBM SPSS version 23.

## Results

### Demographic characteristics

This study enrolled 463 late-referral HD patients, 63.3% male (*n* = 293), with a mean age of 60.4 ± 15.8 years. UK patients were younger with higher rates of glomerulonephritis and diabetes; autosomal dominant polycystic kidney disease (ADPKD) was more frequent in Italian cohort (Table 1).

**Table 1.** Baseline demographic and clinical characteristics of late-referral hemodialysis patients by cohort (UK and Italy).

Characteristics	Overall (*N* = 463)	UK (*N* = 249)	ITA (*N* = 214)	*p*-Value
Age (years)	60.4 ± 15.8	55.7 ± 15.3	65.8 ± 14.8	<0.001
Male	293 (63.3%)	162 (65.1%)	131 (61.2%)	NS
Primary disease				
Glomerulonephritis	74 (16%)	55 (22.1%)	19 (8.9%)	<0.001
Autosomal dominant polycystic kidney	28 (6%)	8 (3.2%)	20 (9.3%)	0.006
Ethnicity				
Black-Caribbean	143 (30.9%)	136 (54.6%)	7 (3.3%)	<0.001
White	290 (62.6%)	87 (34.9%)	203 (94.9%)	<0.001
Other	30 (6.5%)	26 (10.4 %)	4 (1.9%)	<0.001
Medical comorbidities				
PMK	12 (2.6%)	5 (5%)	7 (3.3%)	NS
Immunosuppression	27 (5.8%)	17 (6.8%)	10 (4.7%)	NS
Malignancy	24 (5.2%)	17 (6.8%)	7 (3.3%)	NS
Diabetes mellitus	190 (41%)	122 (48.9%)	68 (31.8%)	0.001
High blood pressure	335 (72.4%)	181 (72.7%)	154 (72%)	NS

PMK: pacemaker carriers; UK: United Kingdom; ITA: Italy.

Values are presented as mean ± standard deviation for continuous variables and as number (percentage) for categorical variables.

The study cohort included 143 Afro-Caribbean patients (30.9%), 290 Caucasians (62.6%), and 30 individuals from other ethnic backgrounds (6.5%; Table 1) with significant differences in ethnic distribution between UK and Italian cohorts (*p* < 0.001; Table 1).

**Figure 1.**
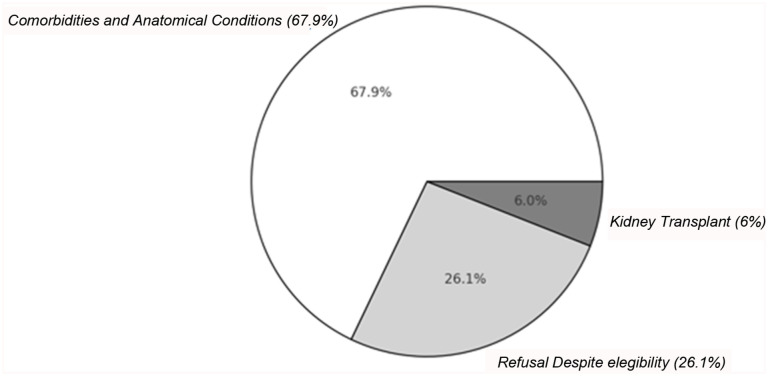
Reasons for AVF non-creation in late-referral hemodialysis patients. Distribution of reasons for AVF non-creation among 268 patients, including refusal despite eligibility (26%).

A total of 195 patients (42.1%) underwent AVF creation following tunneled CVC placement. Median time from CVC placement to AVF creation was 80 days (IQR 18–223), significantly shorter in the Italian cohort (28 days, IQR 1–60) than the UK cohort (140 days, IQR 38–251; *p* = 0.001). AVF creation occurred within 90 days in 51.7% of cases (*n* = 101). AVF creation was significantly higher in the UK (68.6%) than Italy (31.4%; *p* = 4.08E-8). The reason for non-creation of an AVF in 268 patients was: unfavorable vascular anatomy (68%, *n* = 182), imminent kidney transplantation (6%, *n* = 16), or patient refusal despite eligibility (26%, *n* = 70; Figure 1). Refusal was more common in the UK (38.8%, *n* = 45; mean age 53.8 ± 19.1 years) than Italy (16.3%, *n* = 25; mean age 56.2 ± 12.9 years), with no significant demographic or clinical predictors.

### Primary endpoint: All-cause mortality

**Figure 2.**
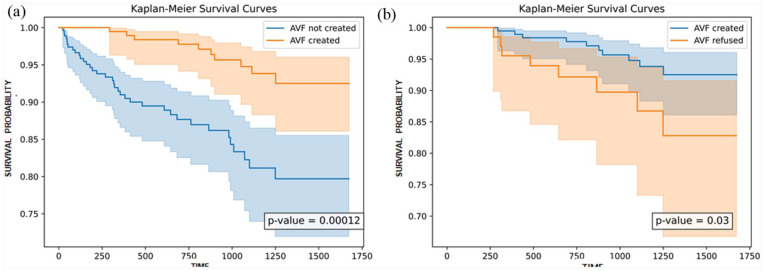
Kaplan-Meier survival analysis by AVF creation status in UK and Italian cohorts: (a) survival curves for all patients, comparing AVF recipients to CVC-dependent patients (*p* = 0.00012) and (b) survival curves for patients, comparing AVF recipients with patients who declined AVF creation despite eligibility (*p* = 0.03).

Overall, mortality prevalence was elevated in CVC-dependent patients compared to AVF recipients (*p* = 0.002). Patients declining AVF creation had higher mortality (11.4% vs 5.1%) and CRBSI rates (21.4% vs 11.8%; *p* = 0.04) compared to AVF recipients, reflecting prolonged CVC dependence. Kaplan-Meier analysis showed a survival advantage for AVF recipients in the overall study population (*p* = 0.00012; Figure 2(a)), and also over eligible patients who declined AVF creation (*p* = 0.03; Figure 2(b)).

In a multivariate model adjusted for age, diabetes, and immunosuppression, only UK cohort patients with AVF creation exhibited improved survival (HR 0.25, 95% CI 0.12–0.55; *p* = 0.001). In a univariate analysis, the time interval between hemodialysis initiation and arteriovenous fistula creation was significantly associated with mortality (OR 1.003, 95% CI 1.000–1.006; *p* = 0.02).

In age-stratified analysis, conversion from CVC to AVF was associated with higher mortality in patients aged ⩾80 years (*p* = 0.06), whereas AVF creation conferred a significant survival advantage in patients <80 years (*p* = 0.0002).

### Secondary endpoint: Vascular access characteristics

**Figure 3.**
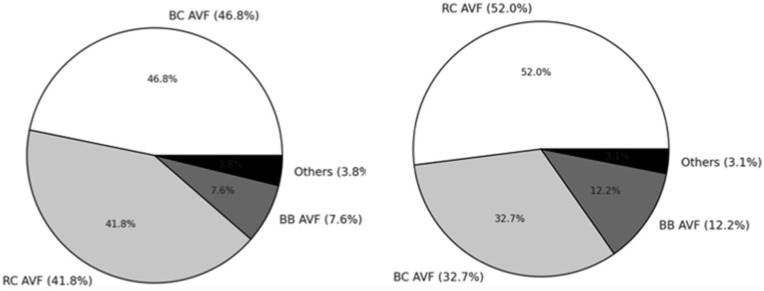
Distribution of arteriovenous fistula (AVF) types by ethnicity and age in late-referral hemodialysis patients: (a) percentage distribution of AVF types in Afro-Caribbean patients and (b) percentage distribution of AVF types in Caucasian patients.

**Figure 4.**
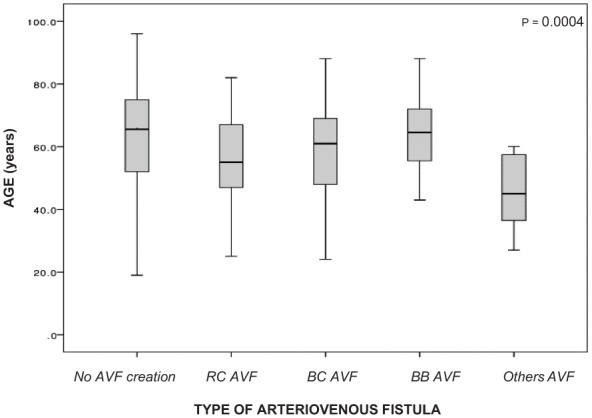
Mean distribution of AVF types by age group. (1) = radiocephalic, (2) = brachiocephalic, (3) = brachiobasilic, (4) = other variants (ulnocephalic, ulnobasilic, endo-radiocephalic), and (0) = no AVF creation.

AVF types included radio-cephalic (20.1%, *n* = 93), brachiocephalic (16.2%, *n* = 75), brachio-basilic (4.3%, *n* = 20), and other variants (1.5%, *n* = 7; e.g. ulno-basilic, ulno-cephalic, endo-radio-cephalic; Table 2). Gender, diabetes mellitus, ADPKD, hypertension, and immunosuppression did not significantly influence AVF type selection. Ethnicity and age were key determinants: radio-cephalic AVFs predominated in Caucasians (17.6%), while brachiocephalic AVFs were more frequent in Afro-Caribbean patients (25.9%; *p* = 0.01; Figure 3(a) and (b)). Younger patients were significantly more likely to receive less common AVF variants (*p* = 0.004; Figure 4). Statistically significant age difference was observed between AVF recipients and non-recipients, with median ages of 57.5 years (IQR 47-68) and 66 years (IQR 52-75), respectively (*p* = 0.0003). A further significant age difference was noted between patients aged ⩾80 years and those <80 years (*p* = 0.0001).

**Table 2.** Arteriovenous fistula creation and non-creation rates in late-referral hemodialysis patients by cohort (UK and Italy).

Category	Overall (*N* = 463)	UK (*N* = 249)	ITA (*N* = 214)	*p*-Value
AVF creation	195 (42.1%)	134 (55.2%)	61 (33.8%)	<0.001
RC AVF	93 (47.7%)	52 (38.8%)	41 (67.2%)	
BC AVF	75 (38.5%)	66 (49.2%)	9 (14.8%)	
BB AVF	20 (10.2%)	9 (6.8%)	11 (18.0%)	
Others	7 (3.6%)	7 (5.2%)	—	
Non AVF creation	268 (57.9%)	115 (44.8%)	153 (66.2%)	NS
Kidney transplantation	16 (6.0%)	10 (8.7%)	6 (3.9%)	NS
Surgery refused	70 (26.1%)	45 (39.1%)	25 (16.3%)	NS
Unfavorable anatomy	188 (67.9%)	60 (52.1%)	122 (79.7%)	<0.001

AVF types include radiocephalic (RC), brachiocephalic (BC), and brachiobasilic (BB). “Others” includes ulno-basilic, ulno-cephalic, and endo-radio-cephalic AVFs. Reasons for non-creation are also detailed. Data are expressed as number (percentage).

### Secondary endpoint: Impact of CVC-to-AVF conversion on AVF patency

Overall primary patency rate at 2, 6, 12, 24 months were: 68.7%, 66.6%, 63.5%, and 60% respectively. Type specific rates for RC-AVF, BC-AVF, BB-AVF, and for other AVFs are reported in Table 3.

**Table 3.** Primary and secondary patency rates at 6, 12, and 24 months.

Patency	RC-AVF (*n* = 93)	BC-AVF (*n* = 75)	BB-AVF (*n* = 20)	Others (*n* = 7)	Overall (*n* = 195)	*p*-Value
6 months						
Primary patency	72% (*n* = 67)	62.7% (*n* = 47)	65% (*n* = 13)	43% (*n* = 3)	66.6% (*n* = 130)	0.04
Secondary patency	73% (*n* = 68)	86.7% (*n* = 65)	90% (*n* = 18)	85.7% (*n* = 6)	88.2% (*n* = 172)	NS
12 months						
Primary patency	67.7% (*n* = 63)	60% (*n* = 45)	65% (*n* = 13	43% (*n* = 3)	63.5% (*n* = 124)	NS
Secondary patency	63.5% (*n* = 59)	77.3% (*n* = 58)	85% (*n* = 17)	85.7% (*n* = 6)	78.5% (*n* = 153)	NS
24 months						
Primary patency	63.5% (*n* = 59)	56% (*n* = 42)	65% (*n* = 13)	43% (*n* = 3)	60% (*n* = 117)	NS
Secondary patency	58% (*n* = 54)	73.3% (*n* = 55)	80% (*n* = 16)	85.7% (*n* = 6)	72.8% (*n* = 142)	NS

Dates are reported as percentages with the corresponding number. A statistically significant difference in primary patency across AVF types was observed at 6 months (*p* = 0.04), whereas no significant differences were found in secondary patency.

Primary patency differed by AVF type at 6 months (*p* = 0.04) only and was age-independent.

At 24 months, primary patency was lower in patients with diabetes than in those without (38.5% vs 61.5%), but the difference was not statistically significant.

Overall, secondary patency rate at 2, 6, 12, 24 months were: 93.9%, 88.2%, 78.5%, 72.8% respectively. Detailed values by AVF type are provided in Table 3.

Salvage procedures before AVF use included re-do anastomosis or proximalization (*n* = 15) and balloon angioplasty (*n* = 26); procedures performed after AVF utilization included open thrombectomy (*n* = 7) and surgical interventions (re-do anastomosis or proximalization (*n* = 10)).

### Secondary endpoint: Catheter-related bloodstream infections

CRBSIs occurred in 74 patients (16.0%), and were sustained by Gram-positive bacteria in 70.3% of the cases (*n* = 52), Gram-negative in 28.4% (*n* = 21), and mixed infections in 1.4% (*n* = 1; Table 4). CRBSI incidence per 1000 CVC-days was 10.1% in Italian cohort and 33.6% in UK cohort (*p* = 0.009).

**Table 4.** Infective complications and management of tunneled CVCs in late-referral hemodialysis patients by cohort.

Category	Overall (*N* = 463)	UK (*N* = 249)	ITA (*N* = 214)	*p* Value
*Infective complications*				
CRBSI	74 (16%)	41 (16.5%)	33 (15.4%)	NS
Gram Pos	52 (11.2%)	32 (12.8%)	20 (9.3%)	
Gram Neg	21 (4.5%)	8 (3.2%)	13 (6%)	
Gram Pos and Neg	1 (0.2%)	1 (0.4%)	—	
Endocarditis	6 (1.3%)	3 (1.2%)	3 (1.4%)	NS
Exit-site infection	7 (1.5%)	7 (2.8%)	—	0.02
Median ΔT (CVC insertion and CRBSIs)	137 (IQR 38–343)	123 (IQR 33–222)	228 (IQR 51–600)	
*Treatment*				
CVC removal	3 (0.6%)	2 (0.8%)	1 (0.5%)	NS
CVC removal and replacement in 24 h	41 (8.9%)	34 (13.7%)	7 (3.3%)	<0.001
CVC exchange	21 (4.5%)	2 (0.8%)	19 (8.9%)	<0.001
Antibiotic ev	12 (2.5%)	7 (2.8%)	5 (2.3%)	NS

Catheter-related bloodstream infections (CRBSIs) are further classified by microbiological isolate: Gram-positive, Gram-negative, or mixed. ΔT refers to the median time (in days) between CVC insertion and onset of CRBSI, reported with interquartile range (IQR). Management strategies include CVC removal, removal and replacement within 24 h, CVC exchange, and intravenous antibiotic therapy. Data are presented as number (percentage), unless otherwise specified.

No significant associations were observed between CRBSI incidence and diabetes or immunosuppression. All six endocarditis cases (1.3%) were linked to Gram-positive CRBSI, and seven patients (1.5%) had concurrent exit-site infections. Management strategies included CVC removal with replacement within 24 h (55.4%, *n* = 41), CVC exchange (28.3%, *n* = 21), removal without replacement (4.1%, *n* = 3), and IV antibiotics alone (16.2%, *n* = 12). Relapse rates were significantly higher in the UK (19.4%, *n* = 8) than in the Italian cohort (0%; *p* = 0.01), with Gram-positive bacteria driving 87.5% of relapses (Table 5). Median time from CVC placement to CRBSI onset was 137 days (IQR 38–343), shorter in the UK (123 days, IQR 33–222) than Italy (228 days, IQR 51–600; *p* = 0.04). Kaplan-Meier analysis confirmed a higher and earlier infection rate in the UK cohort (*p* = 0.02). No significant correlations emerged with gender, age, ethnicity, diabetes, or hypertension.

**Table 5.** CRBSI relapse rates by management strategy in late-referral hemodialysis patients (UK and Italy).

Category	Relapses	GRAM+ relapse	GRAM− relapse
UK			
CVC removal and replacement in 24 h (*N* = 34)	7 (20.5%)	6 (17.6%)	1 (2.9%)
CVC guidewire exchange (*N* = 2)	1 (50%)	1 (50%)	0 (0%)
ITA			
CVC removal and replacement in 24 h (*N* = 7)	0 (0%)	0 (0%)	0 (0%)
CVC guidewire exchange (*N* = 19)	0 (0%)	0 (0%)	0 (0%)

Relapse is defined as recurrence of infection with the same microorganism. Strategies include catheter removal and replacement within 24 h and catheter exchange over a guidewire. Data are expressed as number (percentage) of relapses overall and stratified by Gram-positive and Gram-negative organisms.

## Discussion

This study compares vascular access outcomes among late-referral hemodialysis patients within two distinct healthcare systems.

By analyzing AVF timing, CRBSI, and patient survival, we identify modifiable factors that significantly influence prognosis in this high-risk population.

The type of vascular access remains a key prognostic determinant, with AVFs consistently associated with superior long-term patency, lower infection rates and improved survival compared to tunneled CVC, as supported by prior literature.^4,5,13,21^

Survival Kaplan-Meier analyses were conducted on an intention-to-treat basis from the time of AVF creation; consequently, crossover events (loss of patency or creation of another access) did not reassign patients to a different group, thereby avoiding time-dependent bias. Our observations confirm previous reports showing a significant improvement in mortality of patients initiating hemodialysis with a CVC and converted to AVF compared with those remaining CVC-dependent.^25^ Similarly, Raimann et al.^26^ reported that conversion within 6 months confers a survival benefit.

In our cohort, earlier AVF creation was independently associated with reduced mortality (OR 1.003; *p* = 0.026), further emphasizing the prognostic importance of timely vascular access intervention. Interestingly, Paparella et al.,^27^ in a single-center experience, observed that in elderly patients starting dialysis with a tunneled CVC, vascular access type did not influence mortality and in this setting the only variable associated with patients’ survival were the main comorbidities.

In contrast, another study by Ko et al., suggested that starting dialysis with a CVC followed by AVF creation within 1 year may be acceptable in patients ⩾80 years, compared with starting directly with an AVF.^12^ Nevertheless, our results demonstrated higher mortality among patients ⩾80-years who underwent vascular access conversion (*p* = 0.06), whereas AVF creation was associated with a significant survival benefit in patients <80 years (*p* = 0.0002).

Prolonged CVC dependence, however, was associated with increased CRBSI incidence per 1000 CVC-days, consistent with the known higher mortality risks, with rates of 19.1% in the Italian cohort and 33.6% in the UK cohort (*p* = 0.009).

CRBSI onset occurred earlier in the UK cohort (median 123 days vs 228 days; *p* = 0.04) and was associated with higher relapse rates (19.4% vs 0%; *p* = 0.01).

These findings may reflect differences in who monitors vascular access (namely, dialysis nurses in the UK vs nephrologists in Italy) as well as variability in clinical protocols governing access surveillance and intervention.

Gram-positive bacteria dominated CRBSI aetiology (70.3%), aligning with prior reports,^16^ yet no significant comorbidity associations emerged, indicating that infection risk may hinge more on CVC duration and system-level factors than patient-specific variables.

Despite guideline recommendations,^5,8,18,28^ only 42.1% of late-referral patients transitioned from CVC to AVF. Notably, 26% of AVF-eligible patients declined surgery, with refusal rates markedly higher in the UK (38.8%) than in Italy (16.3%), potentially reflecting differences in patient education or counseling approaches across these two countries.

To address this, structured education programs, incorporating visual aids, risk-benefit discussions, and psychosocial support, could enhance patient acceptance of AVF, mitigating reliance on CVCs and their associated complications.

The UK cohort showed a higher rate of AVF creation (68.6% vs 31.4%; *p* = 4.08E-8) but also a longer median delay from CVC placement to AVF creation (140 days vs 28 days; *p* < 0.001) compared to the Italian cohort. This delay aligns with previous reports of prolonged CVC dependence in certain healthcare systems^6^ and may reflect both structural and cost-related systems differences. In the Italian model, interventional nephrologists manage both AVF planning and creation, enabling timely conversion despite a lower overall procedural volume, thereby reducing cumulative CVC-days and associated costs. Conversely, AVF creation in the UK is performed by vascular and transplant surgeons, achieving higher procedural volumes but longer waiting times due to greater demand and resource constraints. Although delayed, this higher AVF uptake may reduce long-term costs by limiting cumulative catheter exposure and related complications. Importantly, access conversion costs encompass not only CVC-related expenses but also those related to second-access creation and patency-maintenance procedures.^29^

In addition, the higher prevalence of BC-AVF in the UK cohort (49.2% vs 14.8% in Italy) may be explained by both patient-related factors (the larger proportion of Afro-Caribbean patients) and operator-related factors, as AVFs predominantly created by vascular surgeons in UK, consistent with Napoli et al.^30^

Our findings suggest that age strongly influenced vascular access conversion from CVC to AVF. Specifically, younger patients were more likely to receive an AVF (*p* = 0.0003), whereas those ⩾80 years had significantly lower conversion rates (*p* = 0.0001). In general, as noted by Hussein et al.,^31^ differences in vascular access conversion may reflect not only patient-related factors (such as advanced age) but also center-related aspects, including reimbursement policies and surgical resource availability. Importantly, in our cohort no significant association was observed between primary patency and age, indicating that elderly patients did not experience reduced vascular access survival. Although advanced age may represent a barrier to AVF creation, elderly patients who undergo AVF creation achieve vascular access survival comparable to younger individuals.

Although not statistically significant, 24-month primary patency was lower in patients with diabetes than in those without (38.5% vs 61.4%), a difference that may be attributable to diabetes-related endothelial dysfunction.

The apparent stability of primary patency in BB-AVF and in other rare AVF types likely reflects small samples (*n* = 20 and *n* = 7, respectively).

The survival advantage for AVF recipients underscores the urgency of minimizing CVC dependence in late-referral patients. These findings highlight the need for targeted interventions including accelerated referral pathways, multidisciplinary vascular access teams, and patient education programs with psychosocial support, to address gaps in care delivery. Future research should investigate cross-national policy differences and patient-reported barriers to AVF creation, with the aim of informing global best practices in the management of late-referral hemodialysis patients.

## Limitations

This study has several limitations that should be considered when interpreting the findings. (I) missing comorbidity data: data constraints prevented calculation of the Charlson Comorbidity Index, a validated predictor of AVF patency and patient outcomes, potentially underestimating comorbidity impacts; (II) absence of control group: the lack of an early-referral control group limits direct comparisons between referral timings, which could further elucidate late-referral-specific risks; (III) unmeasured confounders: socioeconomic factors, healthcare access disparities, and patient education levels were not assessed, yet these may influence AVF uptake, CRBSI rates, and survival. These gaps open avenues for future research to refine our understanding of late-referral hemodialysis management.

## Conclusions

This study identified a significant association between delayed AVF creation after hemodialysis initiation and increased all-cause mortality in late referral patients.

Mortality was significantly lower among those receiving an AVF shortly after dialysis initiation, underscoring the prognostic value of timely AVF creation.

These findings highlight the negative prognostic impact of prolonged central venous catheter dependence, reinforcing the clinical necessity of early vascular access planning in patients with chronic kidney disease. Nonetheless, even in the absence of pre-dialysis planning, timely conversion from CVC to AVF in eligible patients represents a critical therapeutic opportunity to improve outcomes, emphasizing that while it may be too late to plan, it is not too late to act.

Prioritizing timely AVF creation, supported by multidisciplinary teams and robust patient education, should be a cornerstone of healthcare policies to enhance survival, reduce complications, and alleviate the clinical and economic burden of late-referral hemodialysis.

## Statements

This investigation was conducted as a retrospective, observational two-cohort study. Clinical and demographic data were obtained exclusively through the retrospective review of electronic medical records and surgical logs from both participating institutions. No prospective enrollment, study-specific procedures, additional diagnostics, therapeutic interventions, or patient contract occurred.

Given that the research relied solely on previously collected, routinely documented clinical data, and did not involve any modification of patient care or prospective data acquisition, it did not meet the criteria for human subject research requiring formal review by an ethics committee. The absence of any interventional component or identifiable patient recruitment ensured that the study complied with the relevant ethical standards for retrospective data analyses, and qualified for exemption from institutional ethical approval.
